# Medical School Without Walls: 50 Years of Regional Campuses at Indiana University School of Medicine

**DOI:** 10.1097/ACM.0000000000004940

**Published:** 2022-08-16

**Authors:** Paul M. Wallach, Deborah R. Birnbaum, Elizabeth R. Ryan, Brandon T. Pieczko, Jay L. Hess

**Affiliations:** 1 **P.M. Wallach** is professor of medicine, Dolores and John Read Senior Professorship in Medical Education, executive associate dean, Educational Affairs, and chief academic officer, Indiana University School of Medicine, Indianapolis, Indiana; ORCID: https://orcid.org/0000-0002-7444-5374.; 2 **D.R. Birnbaum** is educational affairs innovations director and Scholarly Concentrations Program director, Indiana University School of Medicine, Indianapolis, Indiana; ORCID: https://orcid.org/0000-0002-4344-6630.; 3 **E.R. Ryan** is associate dean and director, IUSM-Northwest-Gary, and professor of clinical family medicine, Department of Family Medicine, Indiana University School of Medicine, Gary, Indiana; ORCID: https://orcid.org/0000-0001-8680-3741.; 4 **B.T. Pieczko** is digital and special collections librarian, Ruth Lilly Medical Library, Indiana University School of Medicine, Indianapolis, Indiana; ORCID: https://orcid.org/0000-0003-0054-7260.; 5 **J.L. Hess** is dean, Walter J. Daly Professor, professor of pathology and laboratory medicine, and professor of medicine, Indiana University School of Medicine, Indianapolis, Indiana; ORCID: https://orcid.org/0000-0002-5968-4326.

## Abstract

The history of Indiana University School of Medicine (IUSM) dates to 1871, when Indiana Medical College entered into an affiliation with Indiana University in Bloomington to offer medical education. In 1971, the Indiana General Assembly passed a bill to create and fund a distributed model for medical education for which IUSM was responsible, an innovative approach to implementing a statewide medical education program. IUSM became one of the first U.S. medical schools to implement what is today known as a regional medical campus model. This regional medical campus system has permitted IUSM to expand enrollment based on national and local concerns about physician shortages, increase access to care locally, support expansion of graduate medical education, and provide opportunities for research and scholarship by faculty and students statewide. This effort was made possible by partnerships with other universities and health care systems across the state and the support of local community and state leaders. The model is a forward-thinking and cost-effective way to educate physicians for service in the state of Indiana and is applicable to others. This article highlights milestones in IUSM’s 50-year history of regional medical education, describes the development of the regional medical campus model, recognizes significant achievements over the years, shares lessons learned, and discusses considerations for the future of medical education.

The history of Indiana University School of Medicine (IUSM) dates to 1871. Throughout its history, IUSM has benefited from visionary leadership at state, local, school, and university levels. A combination of physician workforce needs, community engagement, desire to serve the state of Indiana, evolution of national expectations for medical education, and collaboration with state government and regional health systems has resulted in the medical school model that IUSM has today. This article highlights milestones in the medical school’s history, describes the development of the regional campus model, recognizes significant achievements over the past 50 years, shares lessons learned, and discusses considerations for the future of medical education.

## History of IUSM

Indiana University’s (IU’s) engagement with medical education began in 1871, when Indiana Medical College entered into an affiliation with IU in Bloomington. This agreement lasted 10 years. ^1^ In 1903, IU created the School of Medicine with a medical department located in Bloomington and a teaching hospital located in Indianapolis with full-time teaching faculty. ^1^ IUSM was among the early adopters of the recommendation of the American Medical Association and the 1910 Carnegie Foundation Report to have a university affiliation with full-time teaching faculty in hospitals. ^1,2^ In 1922, Valparaiso University Medical College closed and IUSM became the only medical school in Indiana. ^1^ In 1959, IUSM began teaching basic science courses in Indianapolis. ^1^

IUSM has a long history of strong collaboration with the state of Indiana and a shared goal of educating generations of physicians to provide health care to the state. Innovation, collaboration, and careful resource allocation helped govern the medical school’s development. IUSM benefited from state and university resources, research grant funding, and philanthropic support. One early milestone was the consolidation of the medical school in Indianapolis in 1957. As the state’s largest city, Indianapolis had a population that could support adequate clinical experiences for students and where sizable hospitals existed or could be developed. IUSM has also benefitted from being the state’s only MD degree–granting school. ^1^

A recurring question of how many physicians should be trained to meet the needs of Indiana residents existed early in IUSM’s history and continues today. Dean John D. Van Nuys (1947–1964) addressed the workforce shortage issue by increasing graduate medical education (GME) positions and sites and clerkships across the state. During Van Nuys’ tenure, the question of building a second medical school was raised in the Indiana General Assembly. Throughout the 1960s, competition was fierce as communities across the state saw a medical school as a way to develop the physician workforce, promote economic development, and share in the prestige that comes with being home to a medical school. In November 1963, the IU Board of Trustees commissioned Booz Allen Hamilton to study medical education in Indiana. The subsequent report recommended expansion of the existing medical school rather than the creation of a second medical school to achieve the best outcomes in terms of ensuring affordability, maintaining the quality of medical education, and meeting the demand for physicians. Despite this recommendation, the cities of Gary, Evansville, South Bend, and Muncie submitted competing proposals to establish a second medical school, all of which failed in the General Assembly. ^1^

Glenn Irwin, MD, was appointed dean in January 1965 and brought continuity and innovative leadership that resulted in the creation of the regional medical campuses (RMCs). In May 1965, IU President Elvis Stahr and Dean Irwin appointed a committee to make recommendations based on several commissioned studies, most notably the Booz Allen Hamilton report. ^1^ In a 1966 article published in the *Journal of Medical Education*, ^3^ Kenneth Penrod (provost of the IU Medical Center) and Dean Irwin outlined a plan for a distributed statewide medical education system based on the following principles:

- Workforce development and training physicians in the communities where they will work rather than exclusively in academic health centers;
- Programmed, on-line instruction, and learning on demand and in person with mentored teaching faculty member experiences;
- Moving some required medical school content to college years;
- A statewide network of teaching hospitals to provide more residency positions and train more physicians; and
- The emerging concepts of continuing medical education (CME) and lifelong learning.

In the late 1960s, Irwin and Penrod’s concept included on-demand distance learning that involved a “high-speed computer with remote terminals, consisting of both an electric typewriter and a cathode ray screen on which a light pencil records; telefacsimile reproduction; remote printout; new forms of data storage and retrieval; and the possibilities of programmed on-line instruction.” ^3(p1031)^ Penrod argued that with such technology in place “it made little difference whether the first two years of medical school were taught a block or fifty miles from the teaching hospital.” ^1(p269)^ Irwin and Penrod’s solution became known as the “Indiana Plan” or “the medical school without walls.” ^3(pp1030,1035)^ and had students start with 1 year (later extended to 2 years) of studying basic sciences and an introduction to clinical medicine at sites outside Indianapolis. The students would then come to Indianapolis for clinical training and finally move throughout the state for residencies that would be expanded. ^3^ In addition, the passing of The Heart Disease, Cancer, and Stroke Amendments of 1965 ^4^ had provided an opportunity for Penrod and Irwin to enact and effectively fund their plan for statewide medical education in partnership with local training sites. This federal legislation established a program for grants “to encourage and assist in the establishment of regional cooperative arrangements among medical schools, research institutions, and hospitals for research training (including continuing education) and for related demonstrations of patient care in the fields of heart disease, cancer, stroke and related diseases.” ^4(p926)^

In 1968, Purdue University and the University of Notre Dame were part of a pilot program to test whether IUSM could educate medical students outside Indianapolis and Bloomington, and whether a statewide network of campuses could work. Three students participated at Purdue and 2 at Notre Dame. With these modest beginnings, several more legislative proposals and considerable political maneuvering followed. In 1969 and 1970, Indiana’s governor formed the Commission on Medical Education, which adopted a resolution to create 7 medical education centers in Lake County (Northwest Campus), South Bend (Notre Dame), Fort Wayne, Muncie, Lafayette (Purdue), Terre Haute, and Evansville. ^1^ IUSM was already operating in Indianapolis and Bloomington. In 1971, the Indiana General Assembly passed House Bill 1439 to create and fund a regional medical system. The Indiana Plan became law, and IUSM was charged with the “orderly development and expansion of medical education program in cooperation with host institutions at the seven centers.” ^1(p273)^ At this time, IUSM was one of the nation’s early medical schools to implement an RMC model. ^5^ The model more fully emerged with the establishment of 35 RMCs in the United States between 1970 and 1979 as a way to increase learner enrollment, diversity, and clinical experiences. ^6^

With the first year of medical education already underway at the Bloomington, Indianapolis, South Bend, and West Lafayette campuses, partnerships with other universities were key to establishing the additional 3 medical education centers in 1 year and remain so today. Within a decade, most regional campuses had added a second year of medical education, with students transferring to Indianapolis for clinical clerkship training. From 1980 to the middle 2000s, the number of RMCs was stable and the IUSM system was largely unchanged. ^7^ Then, in 2006, a report from the Association of American Medical Colleges (AAMC) Center for Workforce Studies warned of looming physician shortages through at least 2025 and issued a call to increase medical school enrollment by 30%. ^8^ In response, IUSM added more students and began third- and fourth-year medical education at the regional campuses. ^1^ By 2015, all 8 regional campuses in the Indiana statewide medical education system were offering all 4 years of medical education (Table 1).

**Table 1:**
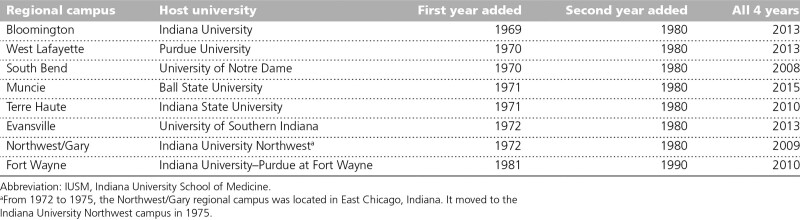
Implementation of Medical School Curriculum at the IUSM Regional Campuses in the Indiana Statewide Medical Education System

Since 2006, regional campus development has been an important strategy nationally to respond to the AAMC expansion request. According to the Liaison Committee on Medical Education (LCME) Annual Questionnaire, ^9^ in academic year 2010–2011, 33% of U.S. AAMC-member medical school respondents reported at least 1 RMC. In 2015–2016, that number increased to 36%. In addition, 16 new RMCs were created, 8 schools expanded already existing campuses, and 15 schools increased the number of students within existing campuses. In academic year 2020–2021, 34% of all U.S. medical schools fully accredited by the LCME have at least 1 RMC. ^9^ Although IUSM has 8 regional campuses, the national average for schools with RMCs is 2.4 campuses. ^10^

## Impact of the Regional Campus Model

The expansion of RMCs has allowed the IUSM to steadily increase enrollment from an average incoming class size of 48 between 1908 and 1913 ^1^ to 215 in 1965, ^11^ 300 in 2000, ^12^ and to 365 in 2021–2022, making IUSM the largest medical school, by enrollment, in the United States. Between 1985 and 2005, regional campus first-year enrollment was approximately 50%. Since that time, regional campus enrollment has increased to 62% (Table 2). According to data from academic year 2020–2021, 15% of third- and fourth-year students are residential at regional campuses, and most students based in Indianapolis complete at least 1 rotation at a regional campus. This expansion of preclerkship education is based on deliberate resource planning and recruitment efforts. IUSM currently has more than 3,000 full- and part-time faculty ^13^ and approximately 3,400 volunteer faculty teaching medical students. ^14^ Overall, more than 5,000 IUSM alumni spent part or all their time at 1 of the 8 campuses other than Indianapolis. ^15^

**Table 2:**
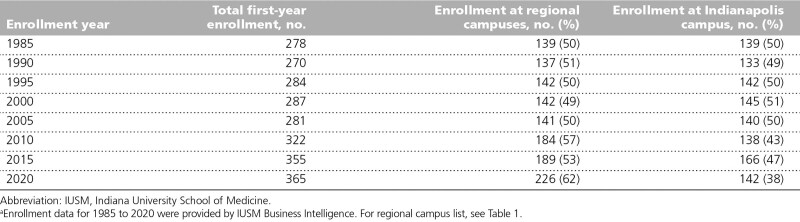
First-Year Medical Student Enrollment at IUSM: Regional Campuses and Indianapolis^a^

Today, IUSM partners with approximately 160 hospitals, health systems, and other health care facilities to provide the full range of clinical experiences and training for students. The regional campus model has been sustained and able to expand through a variety of funding sources, including ongoing state appropriations, tuition, grants, and philanthropy. In 2021, approximately $728,000 in scholarships that donors designated for regional campus students was awarded, and $3 million was awarded to students on regional campuses from all sources, proportionate to overall enrollment. Because all relationships are local, philanthropic giving is the result of sustained partnerships and cultivation efforts between IUSM’s fundraising and philanthropy professionals and regional campus leaders who have relationships in their communities.

Regional campuses have had independent cultures throughout most of IUSM’s history, providing opportunities to pilot new programs and leverage unique aspects and expertise while also requiring efforts to ensure educational comparability across campuses. For example, in the 1970s, the Northwest/Gary campus introduced problem-based learning as a portion of the pedagogy. ^16,17^ Subsequently, problem-based learning has been adopted as a portion of the pedagogic approach for IUSM at all locations. Similarly, for more than a decade, the Terre Haute campus has offered a rural medical education program. This 4-year medical school program includes the core IUSM curriculum with additional emphasis on primary care and the needs of rural communities. The Bloomington campus has a medical sciences program that educates not only medical students seeking an MD degree but also IU undergraduate and graduate students in various programs that lead to bachelor’s, master’s, PhD, and combined degrees. Medical students who remain in Bloomington for their third year are part of a longitudinal integrated curriculum.

Systemwide, all IUSM campuses followed a discipline-based curriculum from the 1970s until 2017, when the curriculum was redesigned into 3 phases, courses were organized by organ system, and modifications were made to introduce more health system science. This redesign acknowledged the importance of training to reflect real-world situations and settings of professional practice and changes in care-delivery models that prepare students to practice medicine in team-based, interdisciplinary settings. The integration of interprofessional, teamwork-based medical education that connects formal knowledge to real-world clinical experiences is one of the recommendations identified in a 2010 report from the Carnegie Foundation for the Advancement of Teaching. ^18^ More recently, simulation and point-of-care ultrasound training have become required curricular elements statewide, and the COVID-19 pandemic accelerated work that was already underway to offer more high-quality, online learning paired with in-person active learning.

Governance and management of the education program are administered through a statewide system that includes statewide course and clerkship teams with representatives from all campuses and accountability up to IUSM’s curriculum steering committee. This structure has provided continuity and comparability across a large, statewide system. It also allowed for rapid adjustments schoolwide necessitated by the COVID-19 pandemic. Other steps have been taken to address challenges of being 1 medical school with 9 campuses. The shift to a single horizontally and vertically integrated curriculum has helped ensure comparability of the educational program statewide. A centralized mentoring and advising program is deployed at each campus as a resource available to all students. Functional integration is fostered through regional campus faculty participation in faculty committees and engagement by members of the dean’s staff and departmental chairs. Within the Office of the Dean, the Faculty Affairs Professional Development and Diversity Office spearheads professional development opportunities for faculty at all ranks, including volunteer faculty. Faculty members across the state have appointments in IUSM departments and are integrated with those departments through regular meetings of statewide clerkship directors with campus faculty and staff members, as well as department all-faculty meetings, annual retreats, and invitations to CME programs. All faculty members also receive newsletters, are involved in data sharing, and participate in regular faculty reviews. In addition, each regional campus was charged with developing its own special area of scholarly concentration that allows for focus, marketing, and the potential for research and scholarship for students, faculty, and staff (described below). With the help of student assessments, program evaluations, learning technology, and input from the teaching and learning community at IUSM, the medical school curriculum continues to evolve and improve to meet the ever-changing training needs of successful physicians.

Residency Match results are also comparable among students at regional campuses and the Indianapolis campus. Between 2008 and 2018, regional campus students matched into highly competitive specialties, such as dermatology, emergency medicine, neurosurgery, vascular surgery, and otolaryngology, at the same rate as students on the Indianapolis campus. ^12^ Early regional campus students cited the closeness they found with their classmates and professors that facilitated learning, and this closeness persists today. ^15^ In recent student satisfaction surveys, regional campus students articulated that they appreciate the close relationships they develop with preceptors during early, hands-on clinical experiences.

## GME Needs and Expansion

One of the compelling reasons for medical school class size expansion through a regional campus model was to increase the number of physicians trained to serve the state of Indiana. As a result, approximately 80% to 85% of an incoming class is composed of Indiana residents. During the past 50 years, the percentage of IUSM graduates who stay in Indiana for residency has fluctuated between 32% and 56% (Table 3). The relatively small number of residency positions per state population impacts the state’s ability to retain medical school graduates in Indiana for residency training. As of December 31, 2018, Indiana had approximately 22 residents and fellows on duty in Accreditation Council for Graduate Medical Education–accredited programs per 100,000 population, ranking 43rd of the 50 states, and only 10 primary care resident physicians per 100,000 population, also ranking 43rd. ^19^

**Table 3:**
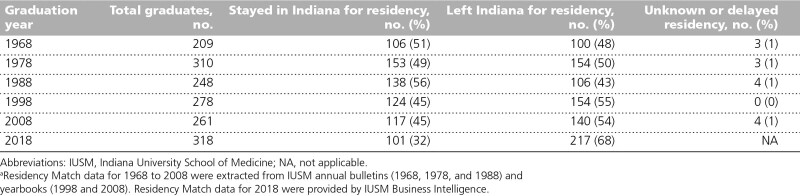
Residency Match Locations for IUSM Graduates: Indiana vs Out of State^a^

GME expansion has been hampered by a cap on the number of training slots at hospitals with existing residencies, with Medicare caps set at the number of positions in place in 1996. Hospitals without residency programs may apply for new Medicare funding and have 5 years to establish and expand their program before a Medicare cap is set. ^20^ Starting new residencies at hospitals without prior residencies has been the centerpiece of strategy for expansion since 2016, with assistance from the State of Indiana. Thus far, new residency programs have included family medicine in Lafayette and internal medicine, family medicine, and psychiatry in Southwest Indiana. The IUSM Northwest campus is starting a psychiatry residency program in 2022–2023, with additional planning underway for residencies in Bloomington. The current expansion programs have already added more than 100 new residency positions in the state, and with planned expansion, IUSM could add more than 200 new residency positions for training physicians in the coming years. For physicians who complete both undergraduate medical education (UME) and GME in a single state, Indiana ranks sixth in retention, with 77.5% staying and practicing in the state. ^19^

IUSM also offers CME to all faculty statewide. CME activity is accredited through a central office and delivered both online and on location throughout the state.

## Research and Scholarship in the Regional Campus Model

Although medical education is comparable across all campuses, the model of scholarship and biomedical research at regional campuses varies. IUSM’s Scholarly Concentrations Program provides a schoolwide structure that shines a spotlight on areas of expertise at each campus in a longitudinal cocurricular experience for students that combines coursework, a scholarly project, and a scholarly product (Table 4). This optional program runs alongside the core medical education curriculum and offers students an opportunity to live, study, and connect deeply in an area of personal interest that enhances their training and competitiveness for residency. In addition to traditional biomedical research topics, such as genetics in medicine, most concentrations allow for community-engaged research and scholarship for both students and faculty. Partnerships extend beyond IUSM departments to other schools, including Indiana University, Purdue University, the University of Notre Dame, and the world-renowned Kinsey Institute. Each regional campus created its own scholarly concentration(s) that aligned with its areas of strength and can be used as a recruitment tool. Since the inception of the Scholarly Concentrations Program in 2019, approximately half of the nearly 390 students enrolled in a scholarly concentration are regional campus students, and two-thirds are participating in their home campus concentration.

**Table 4:**
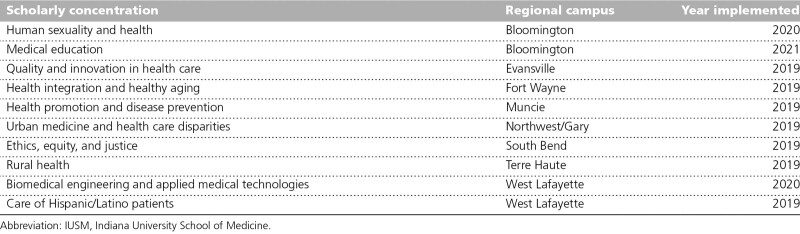
IUSM Scholarly Concentrations Program by Regional Campus

Traditional biomedical research has undergone significant changes in the past 50 years, with the need for increasingly sophisticated and costly research infrastructure and more research being done by teams than by individuals. These changes have impacted IUSM’s strategies for research investment and funding across its campuses (Table 5). In calendar year 2020, IUSM received nearly $438 million in external research funding (excluding West Lafayette/Purdue University funding [see below]). Regional campus research funding comprised approximately 1% of the medical school’s total research funding.

**Table 5:**
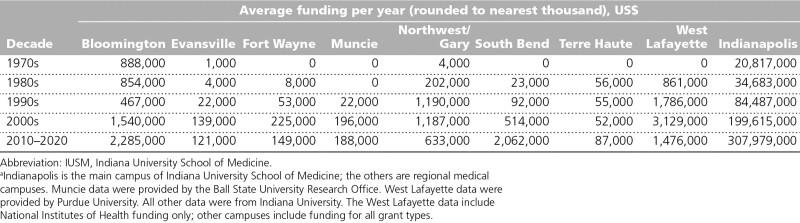
IUSM Campus Annual Research Funding Awards^a^

Over the past 50 years, the primary focus of the regional campuses has been on first- and second-year medical education. The largest research footprints outside Indianapolis are at the regional campus in Bloomington, which has long offered a medical science program, and at the regional campus in West Lafayette, in partnership with Purdue University. The extent of research on the other campuses has waxed and waned and has been largely the result of the regional campus deans’ interests and investigator availability. IUSM is increasingly moving to a more strategic approach to build on the long-term strengths and expertise on campuses through the Scholarly Concentrations Program. In addition, IUSM is committed to ensuring that all students have exposure to the scientific method and have scholarly opportunities available through both the core curriculum and statewide programs, such as the Indiana University Medical Student Program for Research and Scholarship and the Indiana Clinical and Translational Sciences Institute.

## Lessons Learned

Much has been learned from the regional campus model at IUSM during the past 50 years. Our leaders’ vision for creating a distributed medical education model that connects UME, GME, and CME throughout the state using distance technology; governance structure; relationships with the state and local governments; and funding support at the state, local, and philanthropic levels was insightful for the time, and this model helped our school adapt during the COVID-19 pandemic. Each component of the statewide model functions individually, yet the interconnections and relationships of the components make our system work. The following are some lessons we have learned that we believe are valuable for other medical schools with regional campuses or those that may consider this model in the future:

- State and community support are critical. This support includes state funding, local philanthropic support, and connections for programming and opportunities for students; health system relationships; and mutually beneficial local university partnerships to support regional campus operations.
- Student services and resources should be similar across all campuses, including sufficient campus funding and space; adequate numbers of faculty and staff to implement the curriculum and create an academic learning community; and human resources, legal, procurement, technology, and other resources needed to meet LCME accreditation requirements. Each campus should have core services and a faculty and staff model outlined in a strategic plan that is monitored regularly.
- A collaborative governance structure and management model works best with input from throughout the system. For example, IUSM course management teams include a representative from each campus and a statewide leader (who can be from any campus) to develop the goals, objectives, assessments, and intended outcomes for each course by phase based on institutional learning objectives and under the oversight of the curriculum committee. This allows us to take into consideration local nuances while delivering the same curriculum across the state.
- Recruiting and retaining volunteer clinical preceptors for a large system is challenging for many regional campuses. In our experience, engaging a large alumni network assists with faculty recruitment as do strong health system relationships. As new residency programs are deployed in the state, we hope that their graduates will also want to serve as future clinical preceptors.
- Creating areas of emphasis or excellence for each campus allows for a focus for recruitment, as well as esteem and scholarship opportunities for students and faculty. IUSM has used the Scholarly Concentrations Program to allow each RMC to create an area of excellence. In addition, a core strength of regional campus models is their relationships with their local communities. Maximizing this strength allows RMCs and IUSM to address the AAMC’s fourth mission area, community collaborations. ^21^
- The challenges of recruiting and retaining so many faculty members statewide necessitate that faculty recruitment be smart and linked to the strategic needs and scholarly concentration focus for each campus. These faculty members play a key role in creating a robust learning community and home for students on each of the regional campuses.
- Because health care is local, regional campuses can play an important role in residency expansion and, in turn, can ultimately create more opportunities for physicians in communities around the state.

## Future Considerations

Innovation and adaptability remain critical for the future of medical education. Pedagogy will, no doubt, continue to change. Active learning will continue to replace many hours of in-person lectures. It will also be necessary to implement best practices for providing didactic and asynchronous content throughout the curriculum and finding ways to include new content. This new content includes health systems science and content to address health disparities, and diversity, equity, and inclusion in medicine, which have been highlighted so prominently throughout the COVID-19 pandemic. ^22^ IUSM’s educational mission must ensure that, regardless of which campus students receive their training at, graduates are competent to practice medicine in the future while also caring for underserved and vulnerable populations.

It will also be more important than ever for medical students to master new and emerging technologies, such as point-of-care ultrasound ^23^ and telehealth. ^24^ The agile nature of the regional campus model allows for experimentation and piloting initiatives on 1 or 2 campuses before expanding them schoolwide. Interprofessional education will also continue to expand in clinical environments, instead of classrooms. Expanding the continuum of learning opportunities from scholarly concentrations to new dual-degree programs would allow students to learn new skills more in depth, build leadership competencies, and enhance not only their own careers but also their ability to effect much needed changes. To achieve this, requirements for the premedical curriculum likely need to change to alleviate demands on medical education.

Facilities that have been built and renovated, or are being planned, create high-level learning communities where students can engage deeply with each other and faculty. The close engagement of small cohorts on regional campuses has influenced the design of a new academic health center being built on the Indianapolis campus. Before and throughout the COVID-19 pandemic, questions have been raised at IUSM about what types of facilities are necessary to foster learning and the enculturation of trainees into the medical profession and still need investigation.

As 1 school with 9 campuses, IUSM allows for multiple roads to excellence through regional partnerships, cocurricular and service-learning opportunities, and areas of scholarly concentration and research. Because the requirements to secure federal research funding are so significant, regional campuses can consider a different approach and recruit core faculty who dedicate most of their efforts to areas of educational leadership, curriculum design, teaching, assessment, mentoring students, contributing to medical education innovation and best practices, and producing educational scholarship. Our vision for a statewide research mission adds diversity, can vary by campus, and provides students with more opportunities for research and scholarship that can be performed throughout their careers.

The state of Indiana has been a strong partner in GME expansion, having awarded $14 million in funding to support feasibility studies and development of new GME programs by hospitals currently without GME. ^25^ This expansion since 2016 is the result of health care systems joining with IUSM as the sponsoring institution to support the goal of increasing access to care and providing a pipeline for physicians in rural and other underserved communities. The expansion represents an important next step in realizing the vision of the founders of the regional campus system at IUSM. These partnerships between IUSM and health care systems will be key to increasing access to care, meeting workforce needs, and improving health in communities across Indiana. IUSM intends to continue these efforts to improve the efficiency and scope of GME across the state.

## Conclusion

IUSM has graduated approximately 50,000 physicians during its history and today is the largest MD degree–granting medical school in the United States based on enrollment. Approximately 10% of IUSM graduates have spent at least part of their time on a regional campus as the vision of our early leaders for geographically dispersed medical education in Indiana has held firm into the 21st century. IUSM’s ability to expand physician education through its regional campus model remains a statewide effort made possible by partnerships with local universities, statewide and community leaders, and health care systems. The model is an insightful, forward-thinking, and cost-effective way to train physicians to serve the state of Indiana and improve the health of its residents.
